# Diversity and community structure of cyanobacteria and other microbes in recycling irrigation reservoirs

**DOI:** 10.1371/journal.pone.0173903

**Published:** 2017-03-16

**Authors:** Ping Kong, Patricia Richardson, Chuanxue Hong

**Affiliations:** Hampton Roads Agricultural Research and Extension Center, Virginia Tech, Virginia Beach, Virginia, United States of America; National Cheng Kung University, TAIWAN

## Abstract

Recycling irrigation reservoirs (RIRs) are emerging aquatic environments of global significance to crop production, water conservation and environmental sustainability. This study characterized the diversity and population structure of cyanobacteria and other detected microbes in water samples from eight RIRs and one adjacent runoff-free stream at three ornamental crop nurseries in eastern (VA1 and VA3) and central (VA2) Virginia after cloning and sequencing the 16S rRNA gene targeting cyanobacteria and chloroplast of eukaryotic phytoplankton. VA1 and VA2 utilize a multi-reservoir recycling irrigation system with runoff channeled to a sedimentation reservoir which then overflows into transition and retention reservoirs where water was pumped for irrigation. VA3 has a single sedimentation reservoir which was also used for irrigation. A total of 208 operational taxonomic units (OTU) were identified from clone libraries of the water samples. Among them, 53 OTUs (358 clones) were cyanobacteria comprising at least 12 genera dominated by *Synechococcus* species; 59 OTUs (387 clones) were eukaryotic phytoplankton including green algae and diatoms; and 96 were other bacteria (111 clones). Overall, cyanobacteria were dominant in sedimentation reservoirs, while eukaryotic phytoplankton and other bacteria were dominant in transition/retention reservoirs and the stream, respectively. These results are direct evidence demonstrating the negative impact of nutrient-rich horticultural runoff, if not contained, on natural water resources. They also help in understanding the dynamics of water quality in RIRs and have practical implications. Although both single- and multi-reservoir recycling irrigation systems reduce the environmental footprint of horticultural production, the former is expected to have more cyanobacterial blooming, and consequently water quality issues, than the latter. Thus, a multi-reservoir recycling irrigation system should be preferred where feasible.

## Introduction

Agriculture uses 70% of global consumptive water [1], and generates huge quantities of runoff which is a major source of impairments to rivers, lakes and wetlands in the United States (http://water.epa.gov/polwaste/nps/agriculture_facts.cfm) and elsewhere [2–4]. Capture and reuse of irrigation and precipitation runoff is of global significance to water conservation and environmental sustainability by increasing the efficiency of agricultural water use and reducing nonpoint source pollution. These benefits, however, do not come without cost. Such practices potentially recycle and spread plant pathogens from isolated infections to an entire farm and from a single farm infestation to other farms sharing the same water source [5, 6]. Some pathogens may survive better in recycled water than in clean water [7–11]. Water quality may deteriorate, resulting in reduced availability of nutrient elements, underperformance of applied pesticides [12–14], and clogging of irrigation pipelines or emitters by microbial mats [15, 16].

Photosynthetic agents are considered the driving force affecting several water quality parameters in recycling irrigation reservoirs (RIRs) [15–17]. However, the diversity and structure of the phytoplankton community in RIRs is largely unknown. Current research on phytoplankton has focused on non-agricultural freshwater ecosystems. Studies ranged from physical and metabolic capabilities, toxin identification, genome sequencing of cyanobacteria to phytoplankton diversity [18–28]. For example, a recent survey of 46 natural lakes, reservoirs and rivers in Virginia, USA identified more than 300 phytoplankton taxa including cyanobacteria and eukaryotic chlorophytes, diatoms and cryptophytes [28]. Similarly, surveys of five Swedish lakes revealed a linkage between bacterioplankton community composition and environmental variables, providing information on community ecology [20, 21]. While inferring the important roles of these microorganisms in global carbon and nitrogen cycles and the quality of public water supplies, these studies have also provided valuable insights into the ecology and potential applications of these microorganisms [18–28].

RIRs may differ in their phytoplankton community composition from natural water bodies for several reasons. First, RIRs receive irrigation runoff containing residues of fertilizers, pesticides and soil microbes as well as plant debris. Second, RIRs may reconfigure runoff through blending with other water sources, progressive dilution in the same reservoir, or channeling to additional reservoirs which may change the composition of the microbial community.

The primary objective of this study was to determine the complexity of cyanobacteria communities in RIRs and its relation to recycling irrigation system setup and other environmental factors. Eight reservoirs and one adjacent stream at three ornamental crop nurseries were surveyed mid to late spring, the busiest growing season of the year for these production facilities.

## Materials and methods

### Selection and configuration of reservoirs and recycling irrigation systems

**Fig 1:**
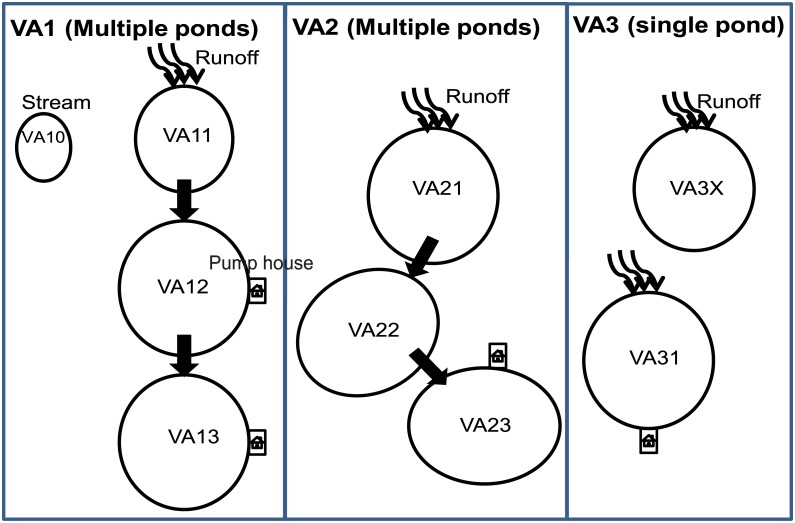
Recycling irrigation systems with single or multiple reservoirs at three ornamental horticultural nurseries surveyed in this study. Arrows illustrate water flow in reservoirs.

Eight reservoirs and one adjacent spring-fed stream at three ornamental crop nurseries in eastern (VA1 and VA3) and central (VA2) Virginia were included in this study. The nursery locations are detailed in Table 1 by latitude and longitude. VA1 and VA2 utilize a multi-reservoir recycling irrigation system with a stepwise water flow while VA3 has a single-reservoir system (Fig 1). At VA1 the first reservoir, labeled VA11, receives irrigation and precipitation runoff directly from ornamental crop production areas. Water flows from VA11 to VA12 through a culvert. When VA12 is full, water can flow into VA13 by opening a sealed culvert. Both VA12 and VA13 are pumped for irrigation. VA10 is a small shaded stream that flows along the perimeter of the nursery; it does not receive any runoff water from the nursery production areas and was occasionally pumped to replenish VA11. Three reservoirs at VA2 (VA21, VA22 and VA23) have a similar water flow arrangement but the top reservoir VA21 has a large holding capacity thus water in this reservoir seldom overflowed into VA22 unless there was a severe storm with heavy rain. VA23 receives water from VA22 through a connecting culvert and is the only reservoir used for irrigation. VA31 and VA3X at nursery VA3 are not connected. VA31 directly receives irrigation and precipitation runoff from production areas at this horticultural facility and is pumped for irrigation to adjacent plots. VA3X receives runoff water from nearby agronomic crop fields outside of the property.

**Table 1:** Physical features and environmental variables of eight reservoirs and one adjacent stream surveyed in this study.

Nursery [Lat, Lon] (Sampling date) ^v^	Reservoir ^w^	Function	Features			Water quality ^x^						Nutrient level (mg/L) ^y^		
			Surface size (ha)	Depth (m)	Light access	Chl (mg/L)	DO (mg/L)	EC (μS/cm)	pH	T (°C)	Turb (NTU)	NH_4_	NO_3_	OrthoP
VA1 [36.7N, -76.6W](4/11/12)	VA10	Stream	<<0.1	0.3	Shaded	0.55	8.6	243.0	5.40	12.3	3.9	0.29	7.42	<0.01
	VA11	Sedimentation	0.1	0.7	Open	24.3	9.1	437.3	6.32	16.4	52.1	5.18	12.07	1.76
	VA12	Transition ^z^	0.8	2.0	Open	32.9	9.5	252.0	6.65	18.1	7.7	2.54	5.17	0.69
	VA13	Retention ^z^	6.1	1.5	Open	2.5	9.9	192.9	7.46	17.6	45.3	0.29	0.46	<0.01
VA2 [37.8N, -77.5W](4/23/13)	VA21	Sedimentation	0.8	4.1	Open	25.1	7.9	122.0	6.52	15.4	41.6	0.03	0.31	0.03
	VA22	Transition	1.6	3.3	Partially shaded	7.05	7.3	267.3	6.73	15.5	6.9	0.06	0.44	<0.01
	VA23	Retention ^z^	0.6	3.5	Partially shaded	5.8	7.1	223	6.07	15.8	7.3	0.21	0.31	0.01
VA3[36.9N, 76.5W](5/1/12)	VA31	Sedimentation ^z^	0.8	1.1	Open	66.8	18.1	332.4	10.10	21.0	21.7	0.06	3.04	0.31
	VA3X	Sedimentation	2.0	3.4	Open	1.2	10.9	379.0	7.91	19.6	0.9	0.29	0.09	<0.01

^v^ locations of nurseries indicated with latitude (Lat) and longitude (Lon).

^w^ Reservoirs coded with “1” in the last digit receive runoff directly from production areas of ornamental crops, VA3X received runoff from production areas of agronomic crops. The two reservoirs in VA3 are not connected. Water flows from VA11 to VA13 and from VA21 to VA23.

^x^ All water quality parameters were recorded at sampling. Chl = chlorophyll; DO = dissolved oxygen; EC = electrical conductivity; T = temperature; Turb = turbidity.

^y^ NO_2_ was not included which was <0.1 mg/L in all reservoirs except VA3X (0.3 mg/L).

^z^ Reservoirs from which water was pumped for irrigation.

### Water sampling and environmental data collection

Environmental variables including reservoir function, physical features, surface water quality and nutrient levels are listed in Table 1. Water quality was measured with a 6600V2-4 Multiprobe Datasonde (YSI Inc., Yellow Springs, Ohio, USA) which was deployed in a buoy anchored at the center of each reservoir for continuous data collection. Water samples were taken using 1 liter bottles 20 cm below the surface in a triangular pattern around the buoy at the same time of day in April or May of 2012 or 2013. Water quality spot logs were recorded simultaneously. The collected samples were placed in a cooler and transported back to the laboratory for analyses. Ten ml aliquots of each water sample were immediately filtered through a 0.22-μm membrane and frozen at -20°C before shipping to Brookside Labs (New Bremen, Ohio, USA) for nutrient analysis. Three replicate measurements/samples were taken from each reservoir or stream.

### DNA extraction, PCR and clone library construction

For DNA extraction, 1-liter samples were centrifuged individually at 12,230 g for 50 min in an Avanti J-26XP (Beckman Coulter, Atlanta, GA, USA). The pellets were collected and the supernatant was filtered through 0.22-μm membranes to catch any remaining microorganisms in the sample. DNA was extracted from the pellet and filters with the Power Water^®^ DNA Isolation kit (MO BIO Laboratories, Inc, Carlsbad, CA, USA). An equal volume of the three replicate DNA samples from the same reservoir was pooled and quantified using a DU 800 Spectrophotometer (Beckman Coulter, Atlanta, GA, USA). 20 ng of each pooled DNA sample was used as template in PCRs with cyanobacteria primers CYA359F and CYA781R [29] to amplify the 16S rDNA genes in cyanobacteria and in chloroplasts of eukaryotic phytoplankton. In addition to the template DNA, the 25-μl reaction contained PCR buffer, 0.5 μl of 10 mM each primer, 1 μl of 25 mM MgCl_2_, 1μl of 2 mM dNTPs, 0.25 μl 100×BSA, 0.5 μl Taq polymerase (TaKaRa Bio Inc, Japan), and dH2O. The reaction was run in an Eppendorf Master Cycler with an initial 2 min denaturation at 94°C, followed by 30 cycles of 30 sec at 94°C, 30 sec at 55°C, and 30 sec at 72°C. PCR products were checked for quantity and size by electrophoresis and ethidium bromide staining.

Clone libraries of the PCR amplicons from each reservoir were generated with the TOPO^®^ TA Cloning Kit (Life Technology-Invitrogen, Carlsbad, CA, USA) according to the manufacturer’s instruction. Over 100 clones per library were randomly selected for sequencing after the 16SrDNA fragment was amplified in the vector with T3 and T7 primers using the same PCR protocol described above. Sequencing was conducted with T3 primer at University of Kentucky’s AGTC sequencing facility.

### Sequence quality control and editing

Poor quality sequences were discarded according to the quality control report from the sequencing facility. The remaining sequences were aligned, reverse complemented and chopped of cloning vector sequences with Mega6 [30]. Chimera sequences were removed with DECIPHER [31] or Mothur software package V.1.33.3. Other sequence processing steps included alignment, screening, filtering, and preclustering with Mothur. Name and group files were created or edited with BioEdit V7.2.5 (http://www.mbio.ncsu.edu/BioEdit/bioedit.html) [32] and Microsoft Excel.

### Construction of reference library for sequence alignment and classification

To construct a reference library for use with Mothur, the taxonomy database (nucleotide) at NCBI (http://www.ncbi.nlm.nih.gov) was searched for “16S rDNA cyanobacteria”. The resulting sequences including accession numbers were retrieved with Batch Entrez and then searched on Silva databases (http://www.arb-silva.de/aligner) and classified using SINA [33]. The taxa were then further screened and filtered with Mothur which resulted in 1161 sequences. These sequences were used to construct template files for taxonomic and phylogenetic analysis of the microbial communities in the reservoirs.

### OTU construction, classification, phylogenetic analysis and taxonomy

Operational taxonomic units (OTUs) were built with Mothur based on a distance matrix of the sequences from all water samples at a pairwise distance cutoff of 0.15 (a percent similarity threshold of 85%). To analyze phylogeny of OTUs, sequences clustered into the OTUs at cutoffs of 0.03 were used as representative OTUs to make a neighbor joining tree.

OTU sequences were classified through Mothur with the constructed cyanobacteria reference library and Arb-Silva-SINA against Silva and Greengenes 16S gene libraries. When identities of these sequences were larger than the default of 70%, their taxa were accepted. Taxa of “unclassified” were predicted based on classification of nearby known taxa in the phylogenetic tree (S1 Fig) and assigned into one of the three groups identified in this study (Table 2).

**Table 2:** Summary of taxa identified from eight reservoirs and an adjacent stream by sequencing the 16S rDNA.

Group	Taxon ^y^	No. of clones	No. of OTUs	Example clone ^z^	Sequence accession #	Sequence reference
Cyanobacteria (CB)	***Arthronema***	2	1	VA1150	KP769657	KJ192051
	***Chamaesiphon***	1	1	VA1064	KP769620	FJ849078
	***Cyanothece***	1	1	VA1118	KP769637	AM710387
	***Dolichospermum***	3	1	VA219	KP769715	EU157991
	***Leptolyngbya***	9	6	VA1190	KP769681	AF218372
	***Microcystis***	3	1	VA2134	KP769709	GQ496077
	*Nostocaceae*	1	1	VA1120	KP769641	HM288179
	***Phormidium***	4	4	VA1182	KP769675	HQ189025
	***Pseudanabaena***	26	6	VA3129	KP769753	AJ007908
	***Synechococcus***	284	15	VA21101	KP769706	FJ763781
	*Unclassified CYA*	23	15	VA1174	KP769669	
	*Xenococcaceae*	1	1	VA1136	KP769652	JN397959
		**358**	**53**			
Eukaryotic phytoplankton (EP)	*Chlorophyta*	133	9	VA1230	KP769690	HQ661193
	*Trebouxiophyceae*	19	3	VA1318	KP769701	JF495289
	*Cryptophyta*	59	11	VA2276	KP769730	EF520521
	*Stramenopila*	98	17	VA1212n	KP769686	JF495301
	*Streptophyta*	49	4	VA2331	KP769739	HQ178730
	*Unclassified EP*	29	15	VA2197	KP769716	
		**387**	**59**			
Other bacteria (OB)	***Armatimonas***	1	1	VA2343	KP769741	AY212628
	***Candidate OD1***	17	15	VA22157	KP769721	AF507684
	***Candidate OP11***	1	1	VA1177	KP769672	AB510996
	***Candidate TM7***	9	9	VA3X60	KP769766	GU120573
	***Candidate MLE1-12***	2	2	VA106	KP769618	EU134274
	***Chloroflexi Ellin6529***	1	1	VA1066	KP769621	HM187036
	***Thermomicrobia***	1	1	VA1184	KP769676	EF157268
	*Unclassified OB*	79	68	VA1373	KP769704	
		**111**	**96**			
**Grand Total**		**856**	**208**			

^y^ Taxa were determined through SINA (v1.2.11) with the least common ancestor (LCA) method based on the taxonomy from Greengenes and Silva at ARB-Silva. Taxa assigned to genus level and below are in bold face.

^z^ Clones are coded by reservoir name (First 4 digits), followed by 2 to 3 digits of its sequential order number in the library.

### Community microbial diversity analysis

Alpha-diversity, an indicator of complexity or richness of the samples, was measured by the rarefaction curve and inverse Simpson index through Mothur. The results were graphed with Microsoft Excel. Beta-diversity (the membership) and microbial community composition as well as their relation to environmental variables were measured with OTU- and taxon-based approaches. For OTU-based analysis, a heat map was generated with the shared file from OTUs of individual reservoirs at cutoffs of 0.03 using Mothur [34] and R version 3.1.0 (http://www.R-project.org/) [35]. The map included the relative abundance of dominant OTUs that contained five or more clones in each reservoir and a dendrogram showing the relationship between the reservoirs. Variations of taxon composition in the reservoirs of single and multiple reservoir systems were investigated with non-metric multidimensional scaling (NMDS) through the vegan “metaMDS” function in R [36]. To analyze correlation between community composition and environmental variables including reservoir features, water quality and nutrient levels (Table 1), the vegan “envfit” function was used to fit the variables into the NMDS ordination.

## Results and discussion

### Taxon groups identified from the reservoirs and stream

A total of 856 quality sequences were obtained from the clone libraries of the eight RIRs and one stream. VA10 had the least (82) and VA31 had the most (107) clone sequences. When classified through Silva 16S rDNA databases and Mothur with the cyanobacteria reference constructed in this study, 41.2% of these clone sequences belonged to cyanobacteria (Table 2) with the remaining classified as eukaryotic phytoplankton (45.1%) and other bacteria (13.3%). Many algae contain chloroplasts that evolved from endosymbiotic cyanobacteria [37, 38] and 16S rDNA primers for cyanobacteria have successfully detected green algae by targeting the chloroplast [39]. All of the identified eukaryotic phytoplankton taxa are listed under the division of cyanobacteria in the current microbial 16S rDNA database. It must be noted that the PCR annealing temperature used in this study was 55°C which is lower than the recommended 60°C for cyanobacteria in the original protocol [29]. These results suggest that these cyanobacterium primers may also detect some bacteria under less stringent PCR conditions. Detection of eukaryotic phytoplankton and other bacteria widens the scope of this study to a limited extent. These detections could be a good starting point in understanding microbial community diversity in RIRs. However, if a study’s aim is to investigate the entire phytoplankton community, these cyanobacteria primers alone would be inadequate. For such studies, diversity-driven genome sequencing or metagenomic methods with primers or barcodes for other groups of phytoplankton would be better utilized.

### Membership of identified groups

**Fig 2:**
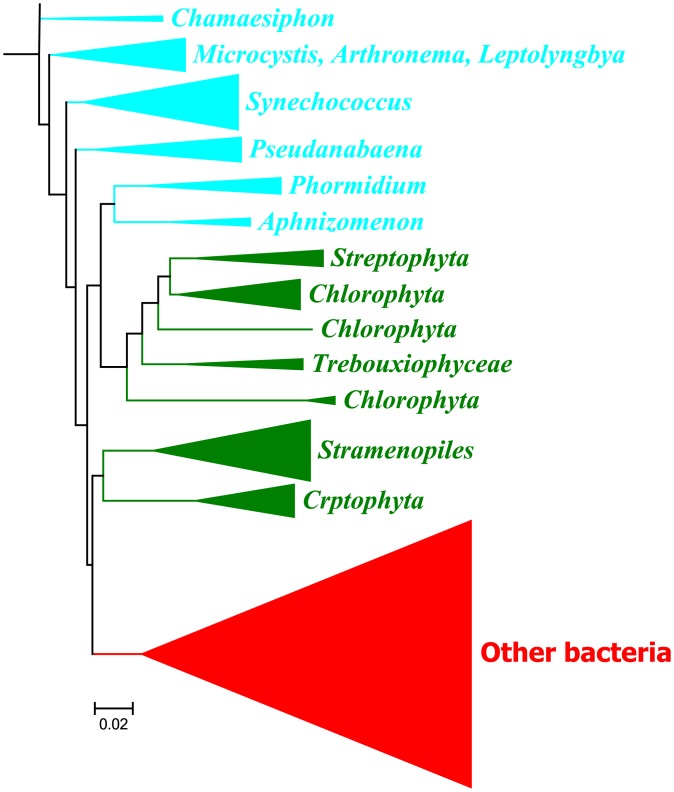
Neighbor joining phylogenetic tree of microbial communities identified from eight reservoirs and one stream surveyed in this study based on 16rDNA amplified with cyanobacteria primers (tree details are shown in S1 Fig). Cyanobacteria, eukaryotic phytoplankton and other bacteria are marked in blue, green and red, respectively.

Because of the limitation of taxon-based classification, operational taxonomy units (OTUs) were used to classify and count the members in the three microbial groups identified. A total of 208 OTUs were identified from 856 clones. Fifty-seven OTUs contained at least two clones (S1 Table). Twenty-two of these OTUs with 5 to 148 clones each hereafter are referred to as dominant OTUs. The remaining 151 OTUs had a single clone. Among them, 36 belonged to cyanobacteria, 81 belonged to other bacteria and 34 belonged to eukaryotic phytoplankton. Phylogenetic relationships among these OTUs are shown in Fig 2 or S1 Fig

Similar numbers of OTUs were assigned to cyanobacteria and eukaryotic phytoplankton, accounting for 25.5 and 28.4% of total clone sequences, respectively (Table 2). There were 53 OTUs in the cyanobacterial group with *Synechococcus* as the most dominant, accounting for 28.3% (79.3% of clones) (Table 2). The 2^nd^ to 4^th^ dominant genera were *Pseudanabaena* (11.3% of OTUs and 7.3% of clones), *Leptolyngbya* (11.3% of OTUs and 2.5% clones) and *Phormidium* (7.5% of OTUs and 1% of clones), respectively. Other genera detected included *Arthronema*, *Chamaesiphon*, *Cyanothece*, *Dolichospermum* and *Microcystis* with much fewer OTUs. However, 28.3% of OTUs in the cyanobacteria group remain unclassified with only 81.6% to 93.3% sequence similarities to those in the Silva 16S rRNA database (S1 Table).

Fifty-nine OTUs were identified in the eukaryotic phytoplankton group. Among them, *Stramenopiles* including diatom, golden algae, brown algae, yellow-green algae and oomycetes were predominant, accounting for 28.8% OTUs and 25.3% clones in the group (Table 2). *Chlorophyta* or green algae including *Trebouxiophyceae* were the second most dominant with 20.3% OTUs and 39.3% clones. *Cryptophyta*, single-celled small algae with two flagella, were the third most dominant taxa with 18.6% OTUs and 15.2% clones. Like cyanobacteria, 26% OTUs of eukaryotic phytoplankton were unclassified.

Other bacteria accounting for 46.2% of total clone sequences included 96 OTUs, almost twice as many as the other two groups. In addition, the percentage of unclassified OTUs was also significantly higher. 99% of these taxa including *Candidate* (97.3% clones) were not classified (Table 2), however some of them such as MLE1-12 or Melainabacteria have recently been assigned to cyanobacteria [40]. This suggests that other bacteria may be important sources of microbial biodiversity in RIRs, and the high rates of unknown taxa in all three groups may reflect uniqueness of microbial communities in RIRs.

### Complexity and structure of microbial communities

**Fig 3:**
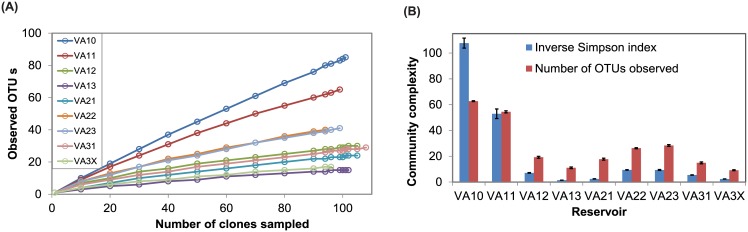
α-diversity of microbial communities in eight reservoirs and one adjacent stream. **A**. Rarefaction curves of operational taxonomic units (OTUs) in clone sequences among the reservoirs. **B**. Inverse Simpson diversity index and number of observed OTUs in the reservoirs.

Community variations were observed among RIRs. Rarefaction curves based on observed OTUs and detected clone sequences in the communities showed relatively higher diversity in VA10 and VA11 than other reservoirs (Fig 3A). Similar results were obtained when the diversity was measured with inverse Simpson index (Fig 3B). VA10 had the highest index, VA11 had the next highest index, followed by VA23, VA22, VA12, VA31, VA21, VA 3X and VA13. VA10 is a stream that never receives runoff from the production nursery and was occasionally pumped to replenish VA11 during the growing season. Thus, this study provides direct evidence on the negative impact of nutrient-rich agricultural runoff on microbial diversity in these aquatic ecosystems.

**Fig 4:**
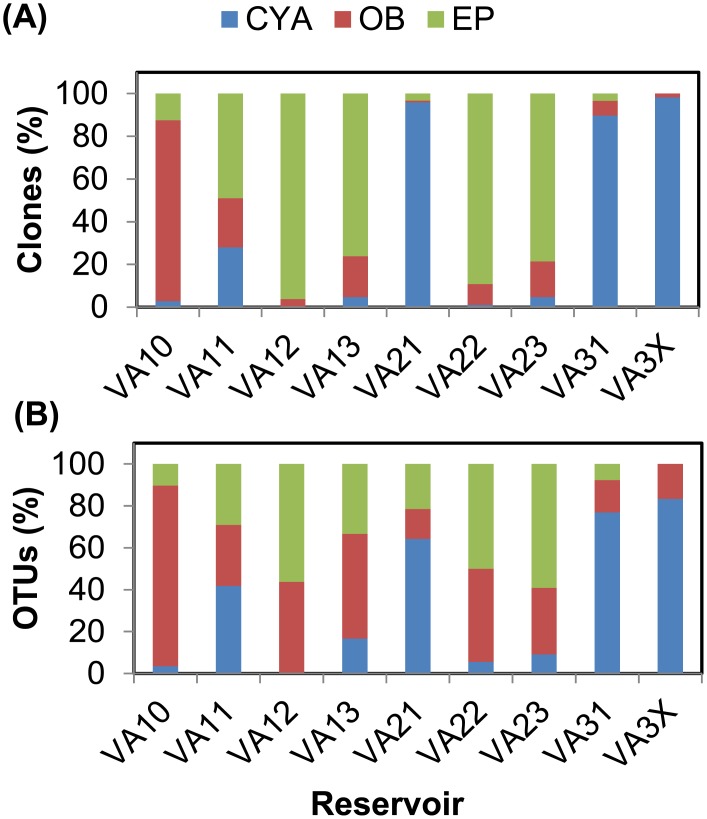
Microbial community composition in eight reservoirs and one adjacent stream. **A.** Evenness or percentage of clones within groups in each reservoir. **B.** Richness or the percentage of OTUs within groups in each reservoir. CYA = cyanobacteria, EP = eukaryotic phytoplankton, and OB = other bacteria.

There was a general association between microbial community composition and reservoir function (Table 1, Fig 4). First, sedimentation reservoirs (VA11, VA21, VA31 and VA3X) had the highest percentage of cyanobacteria clones (Fig 4A) and OTUs (Fig 4B) among the three groups. Second, transition/retention reservoirs (VA12, VA13, VA22 and VA23) were predominantly eukaryotic phytoplankton. Third, VA10, the only runoff-free water was predominantly other bacteria. Multi-reservoir water recycling systems and blending with water from streams, rivers or wells may be effective options to avoid irrigation water quality deterioration resulting from cyanobacterial blooms.

**Fig 5:**
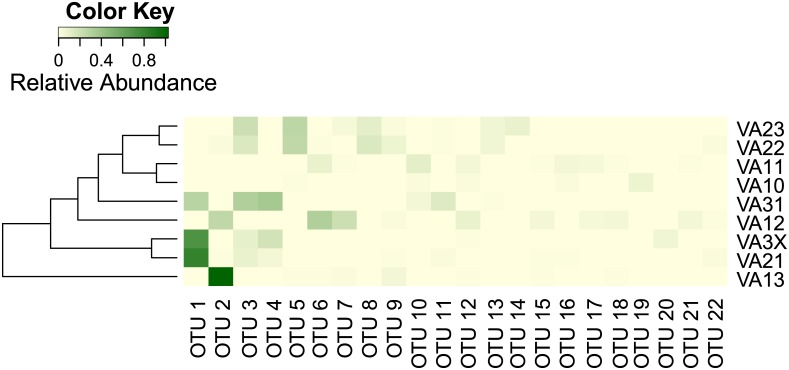
Heat map showing distribution and abundance of 22 dominant OTUs in eight reservoirs and one adjacent stream.

There were also site-specificities within the dominant microbial groups. For cyanobacterium-rich sedimentation reservoirs, *Synechococcus* (OTU1, OTU3 or OTU4) were detected in VA21, VA31 and VA3X but not in VA11 where *Pseudanabaena* (OTU10) was predominant (Fig 5, S1 Table). Similarly, for eukaryotic phytoplankton-rich transition/irrigation reservoirs, few OTUs were present in both VA1 and VA2. VA13 and V12 of VA1 were predominantly *Stramenopiles* (OTU2), while inVA22 and 23 of VA2 several OTUs of *Cryptophyta* were predominant. These site-specificities are likely due to the differences in local soil type, weather conditions and horticultural practices including application of fertilizers, pesticides and plant growth regulators.

Some taxa seemed to have moved along with runoff movement from sedimentation to other reservoirs at nurseries VA1 and VA2. For example, at VA1, *Stramenopiles* (OTU2, 12, and 17) and *Chlorophyta* (OTU 6) were detected in both sedimentation reservoir VA11 and transition reservoir VA12; *Stramenopiles* carried over into the irrigation reservoir VA13 (Fig 5). Similarly, at VA2, the predominant *Synechococcales* (OTU 3) in VA21 was also detected in VA22 and VA23. Whether these newcomers in transition/irrigation reservoirs become dominant over time is not known at this time.

### Ordination of microbial diversity with environmental variables

**Fig 6:**
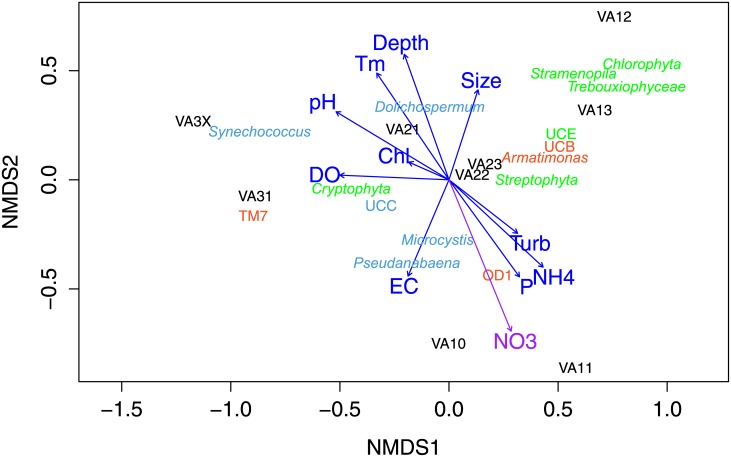
Non-metric Multidimensional Scaling (NMDS) ordination of the variation of taxon compositions in the reservoirs and fitted levels of environmental variables. Direction of the most rapid changes in environmental variables is shown with pointed arrows. The length of the arrows indicates a proportion to the correlation between ordination and environmental variable. Taxa in green, blue and red are eukaryotic phytoplankton, cyanobacteria and other bacteria, respectively. UCC = unclassified cyanobacteria, UCE = unclassified eukaryotic phytoplankton, UCB = unclassified bacteria. Arrow line in purple indicates that the parameter (NO_3_) significantly varied and correlates with the ordination configuration (*P* < 0.1) in permutation tests. Chl = chlorophyll a, DO = dissolved oxygen, EC = electrical conductivity, P = orthophosphate, Tm = temperature, Turb = turbidity.

Reservoir features, water quality and nutrient levels (Table 1) were fitted into the community NMDS ordination to determine whether and how environmental variables may affect microbial diversity. Among the 11 investigated environmental variables, nitrate load (NO_3_^−^) was the only one substantially influencing the ordination (*P* < 0.055) (Table 3), notably in the presence of bacterial *Candidate* OD1, Parcubacteria, a group favoring anoxic environments (Fig 6). Other variables had relatively lower impacts, and chlorophyll level was least significant. Among these low impact variables, pH, temperature and depth positively associated with the dominance of *Synechococcus* and *Dolichospermum* and EC negatively associated with the dominance of *Microcystis* and *Pseudanabaena* (Fig 6). These findings are consistent with the previous observations that cyanobacteria are tolerant of high pH and temperature [41] and that *Synechococcus* grows well under low light [42]. The majority of *Synechococcus* were found in VA21 and VA3X which had relatively deeper water columns (Table 1). This also explains the lack of *Synechococcus* in the VA1 communities where reservoirs were shallow at two meters or less.

**Table 3:** Significance of environmental variables in eight reservoirs and an adjacent stream determined with permutation tests in fitted Non-metric Multidimensional Scaling (NMDS) ordination (Stress = 0.0698).

Environmental variables	NMDS1	NMDS2	r2	*P* (> r) ^X^
Surface size (ha)	0.308	0.952	0.208	0.532
Depth (m)	-0.336	0.942	0.414	0.224
Chlorophyll (mg/L)	-0.915	0.403	0.045	0.871
Electrical conductivity (μS/cm)	-0.392	-0.920	0.255	0.388
Dissolved oxygen (mg/L)	-0.999	0.042	0.278	0.454
pH	-0.857	0.516	0.406	0.228
Temperature (°C)	-0.561	0.828	0.387	0.253
Turbidity	0.789	-0.615	0.177	0.529
NH4 (mg/L)	0.734	-0.680	0.384	0.232
NO3 (mg/L)	0.380	-0.925	0.624	0.055*
OrthoP (mg/L)	0.588	-0.809	0.335	0.322

^X^ Significance * <0.1 based on 1000 permutations. r = squared correlation coefficient

Detection of eukaryotic phytoplankton and other bacterial species may be associated with some other environmental variables. For example, *Chlorophyta*, *Stramenopile* and *Trebouxiophyceae* dominated VA13, the largest reservoir surveyed in this study. *Cryptophyta* dominance was positively associated with DO level, as was the dominance of *Streptophyta* with turbidity, ammonium and orthophosphate. These associations might be indicative of the impact of environmental variables on RIR microbial diversity. Further investigations into whether and how these associations may become significant with time are warranted.

## Conclusions

This study characterized the diversity and composition of cyanobacteria and other detected microbes in RIRs at three ornamental crop nurseries and their relation to reservoir function and environmental variables. The diversity of cyanobacteria detected in RIRs is quite different from that of natural lakes and rivers [28]. This difference highlights the importance of investigation into microbial diversity and population dynamics in RIRs, emerging aquatic ecosystems of global significance.

Within the recycling irrigation systems investigated in this study, cyanobacteria was found dominant in the sedimentation reservoirs, whereas eukaryotic phytoplankton were dominant in the transition and retention reservoirs, and other bacteria were dominant in the runoff-free stream. These results are direct evidence that nutrient-rich horticultural runoff, if not captured and retained on the production properties, will negatively influence microbial diversity in natural water resources including lakes, streams and rivers. Practically, both single- and multi-reservoir recycling irrigation systems reduce the environmental footprint of horticultural production; the former is expected to have more cyanobacterial blooming, consequently water quality issues, in RIRs than the latter. Thus, a multi-reservoir recycling irrigation system is preferred where feasible. Further investigations into how microbial community diversity and structure in RIRs may change seasonally are warranted.

## Supporting information

S1 Fig Expanded neighbor joining phylogenetic tree of OTUs identified by the 16S rDNA sequence from eight reservoirs and one adjacent creek surveyed in this study. (PDF)

S1 Table Summary of OTUs identified by the 16S rDNA sequence from water samples collected from nine reservoirs on three ornamental horticultural nurseries in eastern and central Virginia. (DOCX)
