# Supplementary figures and images for: Diversity and community structure of cyanobacteria and other microbes in recycling irrigation reservoirs
Source: PLoS One. 2017 Mar 16;12(3):e0173903. doi: 10.1371/journal.pone.0173903 (PMC5354426; doi:10.1371/journal.pone.0173903)

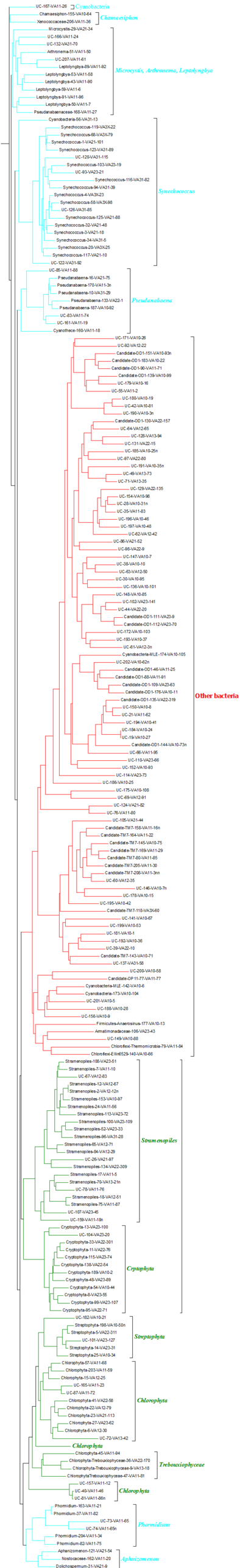

Supplement: S1 Fig — (PDF) [file pone.0173903.s001.pdf]
