# Supplementary material for: Diversity and community structure of cyanobacteria and other microbes in recycling irrigation reservoirs
Source: PLoS One. 2017 Mar 16;12(3):e0173903. doi: 10.1371/journal.pone.0173903 (PMC5354426; doi:10.1371/journal.pone.0173903)
Supplement: S1 Table — (DOCX) [file pone.0173903.s002.docx]

**S1 Table.**  Summary of OTUs identified by the 16S rDNA sequence from water samples collected from nine reservoirs on three ornamental horticultural nurseries in eastern and central Virginia

| **OTU** | **# clones** | **Classification ^X^** | | | **Represent** | **Accession #** | **Identity (%)** |
| --- | --- | --- | --- | --- | --- | --- | --- |
|  |  | **Community type** | **Division** | **Genus ^Y^** |  |  |  |
| 1 | 148 | CYA | Synechococcales | Synechococcus | VA21101 | KP769706 | 99.7 |
| 2 | 108 | EP | Stramenopiles |  | VA1212n | KP769686 | 96.2 |
| 3 | 70 | CYA | Synechococcales | Synechococcus | VA2118 | KP769708 | 99.7 |
| 4 | 48 | CYA | Synechococcales | Synechococcus | VA3X23 | KP769764 | 99.4 |
| 5 | 45 | EP | Streptophyta |  | VA22311 | KP769725 | 99.0 |
| 6 | 35 | EP | Chlorophyta |  | VA1230 | KP769690 | 95.2 |
| 7 | 23 | EP | Stramenopila |  | VA1110 | KP769634 | 99.4 |
| 8 | 21 | EP | Cryptophyta |  | VA2255 | KP769727 | 94.6 |
| 9 | 15 | EP | Chlorophyta | Trebouxiophyceae | VA1318 | KP769701 | 88.6 |
| 10 | 15 | CYA | Pseudanabaenales | Pseudanabaena | VA3129 | KP769753 | 99.4 |
| 11 | 15 | EP | Cryptophyta |  | VA2276 | KP769730 | 96.2 |
| 12 | 15 | EP | Stramenopila |  | VA1267 | KP769696 | 99.4 |
| 13 | 11 | EP | Cryptophyta |  | VA23100 | NA | 98.7 |
| 14 | 7 | EP | Streptophyta |  | VA2331 | KP769739 | 97.1 |
| 15 | 7 | EP | Chlorophyta |  | VA1225 | KP769688 | 96.8 |
| 16 | 7 | CYA | Pseudanabaenales | Pseudanabaena | VA2175 | KP769712 | 98.4 |
| 17 | 6 | EP | Stramenopila |  | VA115 | KP769656 | 86.9 |
| 18 | 6 | EP | Stramenopila |  | VA1251 | KP769694 | 88.4 |
| 19 | 6 | OB | UCB | UCB | VA1027 | NA | 91.3 |
| 20 | 5 | CYA | Pseudanabaenales | Synechococcus | VA3X25 | KP769765 | 99.0 |
| 21 | 5 | OB | UCB | UCB | VA1162 | NA | 90.0 |
| 22 | 5 | EP | Chlorophyta |  | VA1279 | KP769698 | 99.0 |
| 23 | 4 | EP | Chlorophyta |  | VA21113 | KP769707 | 97.5 |
| 24 | 3 | EP | Stramenopila |  | VA1156 | KP769658 | 98.1 |
| 25 | 3 | EP | Streptophyta |  | VA1034 | KP769609 | 100.0 |
| 26 | 3 | EP | UCE | UCE | VA2197 | KP769716 | 87.7 |
| 27 | 3 | EP | Chlorophyta |  | VA2362 | KP769744 | 93.3 |
| 28 | 3 | OB | UCB | UCB | VA1031n | NA | 85.8 |
| 29 | 3 | CYA | Chroococcales | Microcystis | VA2134 | KP769709 | 99.7 |
| 30 | 3 | OB | UCB | UCB | VA1095 | KP769630 | 84.5 |
| 31 | 3 | CYA | Nostocales | Dolichospermum | VA219 | KP769715 | 99.4 |
| 32 | 3 | CYA | Synechococcales | Synechococcus | VA2148 | KP769710 | 98.1 |
| 33 | 3 | EP | Cryptophyta |  | VA22301 | KP769723 | 95.9 |
| 34 | 2 | CYA | Synechococcales | Synechococcus | VA315 | KP769756 | 97.8 |
| 35 | 2 | OB | UCB | UCB | VA1183 | NA | 85.8 |
| 36 | 2 | EP | Chlorophyta | Trebouxiophyceae | VA22170 | KP769722 | 89.5 |
| 37 | 2 | CYA | Oscillatoriales | Phormidium | VA1182 | KP769675 | 98.7 |
| 38 | 2 | OB | UCB | UCB | VA1010 | NA | 86.8 |
| 39 | 2 | OB | UCB | UCB | VA2210 | KP769719 | 87.9 |
| 40 | 2 | EP | Chlorophyta |  | VA1146 | KP769655 | 99.4 |
| 41 | 2 | EP | Chlorophyta |  | VA2258 | KP769728 | 96.5 |
| 42 | 2 | OB | UCB | UCB | VA1081 | NA | 82.5 |
| 43 | 2 | CYA | Pseudanabaenales | Leptolyngbya | VA1190 | KP769681 | 100.0 |
| 44 | 2 | OB | UCB | UCB | VA2220 | NA | 86.5 |
| 45 | 2 | EP | Chlorophyta |  | VA1194 | KP769684 | 88.3 |
| 46 | 2 | OB | Candidate | OD1-ZB2 | VA1125 | KP769646 | 95.6 |
| 47 | 2 | EP | Chlorophyta | Trebouxiophyceae | VA1181 | KP769674 | 99.7 |
| 48 | 2 | EP | Cryptophyta |  | VA2389 | KP769749 | 95.6 |
| 49 | 2 | OB | UCB | UCB | VA1373 | KP769704 | 83.1 |
| 50 | 2 | CYA | Pseudanabaenales | Leptolyngbya | VA117 | KP769666 | 99.4 |
| 51 | 2 | CYA | Pseudanabaenales | Arthronema | VA1150 | KP769657 | 95.9 |
| 52 | 2 | EP | Stramenopila |  | VA2333 | KP769740 | 98.7 |
| 53 | 2 | CYA | Pseudanabaenales | Leptolyngbya | VA1158 | KP769659 | 97.5 |
| 54 | 2 | EP | Cryptophyta |  | VA1044 | KP769613 | 94.6 |
| 55 | 2 | OB | UCB | UCB | VA112 | KP769640 | 89.6 |
| 56 | 2 | CYA | Cyanobacteria | Cyanobacteria | VA3113 | KP769751 | 89.2 |
| 57 | 2 | EP | Chlorophyta |  | VA1168 | KP769665 | 99.7 |
| 58 | 1 | CYA | Synechococcales | Synechococcus | VA3X98 | KP769768 | 96.5 |
| 59 | 1 | CYA | Pseudanabaenales | Leptolyngbya | VA116 | KP769661 | 93.3 |
| 60 | 1 | OB | UCB | UCB | VA1235 | KP769691 | 93.0 |
| 61 | 1 | OB | UCB | UCB | VA123n | KP769692 | 81.3 |
| 62 | 1 | OB | UCB | UCB | VA1242 | NA | 82.1 |
| 63 | 1 | OB | UCB | UCB | VA1250 | KP769693 | 86.8 |
| 64 | 1 | OB | UCB | UCB | VA1265 | KP769695 | 88.1 |
| 65 | 1 | EP | Stramenopila | Stramenopiles | VA1271 | KP769697 | 94.0 |
| 66 | 1 | OB | UCB | UCB | VA1195 | NA | 90.6 |
| 67 | 1 | EP | UCE | UCE | VA1283 | KP769699 | 90.2 |
| 68 | 1 | CYA | Synechococcales | Synechococcus | VA3X79 | KP769767 | 96.5 |
| 69 | 1 | OB | UCB | UCB | VA1291 | KP769700 | 80.1 |
| 70 | 1 | EP | Stramenopila |  | VA1321n | KP769702 | 86.9 |
| 71 | 1 | OB | UCB | UCB | VA1335 | NA | 82.8 |
| 72 | 1 | EP | UCE | UCE | VA1342 | KP769703 | 85.4 |
| 73 | 1 | EP | UCE | UCE | VA1165 | KP769663 | 84.2 |
| 74 | 1 | EP | UCE | UCE | VA1165n | KP769664 | 89.3 |
| 75 | 1 | EP | Stramenopila |  | VA1187 | KP769679 | 87.8 |
| 76 | 1 | OB | UCB | UCB | VA1180 | KP769673 | 73.6 |
| 77 | 1 | OB | Candidate | OP11(WCHB1-64-d153) | VA1177 | KP769672 | 86.1 |
| 78 | 1 | EP | UCE | UCE | VA1176 | KP769671 | 83.5 |
| 79 | 1 | OB | Chloroflexi | Thermomicrobia-JG30-KF-CM45 | VA1184 | KP769676 | 99.7 |
| 80 | 1 | OB | Candidate | TM7-1 | VA1185 | KP769677 | 96.8 |
| 81 | 1 | EP | UCE | UCE | VA1186n | KP769678 | 99.4 |
| 82 | 1 | CYA | Oscillatoriales | Phormidium | VA1175 | KP769670 | 100.0 |
| 83 | 1 | CYA | UCC | UCC | VA1174 | KP769669 | 93.3 |
| 84 | 1 | EP | Stramenopila |  | VA1229 | KP769689 | 96.2 |
| 85 | 1 | CYA | UCC | UCC | VA1188 | KP769680 | 89.2 |
| 86 | 1 | OB | UCB | UCB | VA2152 | KP769711 | 87.7 |
| 87 | 1 | EP | UCE | UCE | VA1172 | KP769668 | 91.4 |
| 88 | 1 | OB | Candidate | OD1-ZB2 | VA1191 | KP769682 | 97.2 |
| 89 | 1 | CYA | Pseudanabaenales | Leptolyngbya | VA1192 | KP769683 | 88.0 |
| 90 | 1 | OB | Candidate | OD1 | VA1171 | KP769667 | 86.8 |
| 91 | 1 | CYA | Pseudanabaenales | Leptolyngbya | VA1196 | KP769685 | 98.7 |
| 92 | 1 | OB | UCB | UCB | VA1222 | KP769687 | 84.7 |
| 93 | 1 | CYA | UCC | UCC | VA2321 | KP769738 | 93.3 |
| 94 | 1 | CYA | Synechococcales | Synechococcus | VA3139 | KP769754 | 95.7 |
| 95 | 1 | EP | Cryptophyta |  | VA2271 | KP769729 | 96.5 |
| 96 | 1 | EP | Stramenopila |  | VA3128 | KP769752 | 99.1 |
| 97 | 1 | OB | UCB | UCB | VA2280 | KP769731 | 88.4 |
| 98 | 1 | OB | UCB | UCB | VA229 | KP769732 | 83.9 |
| 99 | 1 | EP | Cryptophyta |  | VA23107 | KP769733 | 92.4 |
| 100 | 1 | EP | Stramenopila |  | VA23109 | KP769734 | 98.7 |
| 101 | 1 | EP | UCE | UCE | VA23127 | KP769735 | 92.7 |
| 102 | 1 | OB | UCB | UCB | VA23141 | NA | 83.9 |
| 103 | 1 | CYA | Synechococcales | Synechococcus | VA2319 | KP769736 | 95.6 |
| 104 | 1 | EP | UCE | UCE | VA2320 | KP769737 | 91.1 |
| 105 | 1 | OB | UCB | UCB | VA3144 | KP769755 | 88.6 |
| 106 | 1 | OB | Armatimonadales | Armatimonas | VA2343 | KP769741 | 96.8 |
| 107 | 1 | EP | UCE | UCE | VA2345 | KP769742 | 88.1 |
| 108 | 1 | EP | Stramenopila |  | VA2351 | KP769743 | 98.1 |
| 109 | 1 | OB | Candidate | OD1 (ZB2) | VA2363 | KP769745 | 96.2 |
| 110 | 1 | OB | UCB | UCB | VA2366 | KP769746 | 84.3 |
| 111 | 1 | OB | Candidate | OD1 (ZB2) | VA239 | NA | 85.8 |
| 112 | 1 | OB | Candidate | OD1 (ZB2) | VA2370 | NA | 86.8 |
| 113 | 1 | EP | Stramenopila | Stramenopiles | VA2372 | KP769747 | 99.4 |
| 114 | 1 | OB | UCB | UCB | VA2373 | NA | 86.5 |
| 115 | 1 | EP | Cryptophyta | Cryptophyta | VA2374 | KP769748 | 94.9 |
| 116 | 1 | CYA | Synechococcales | Synechococcus | VA3182 | KP769759 | 92.5 |
| 117 | 1 | CYA | Synechococcales | Synechococcus | VA2110 | KP769705 | 98.4 |
| 118 | 1 | OB | Candidate | TM7-3-EW055 | VA3X60 | KP769766 | 95.6 |
| 119 | 1 | CYA | Synechococcales | Synechococcus | VA3X22 | KP769763 | 95.3 |
| 120 | 1 | CYA | UCC | UCC | VA31115 | KP769750 | 88.3 |
| 121 | 1 | CYA | Nostocales | Aphanizomenon | VA2154 | NA | 99.1 |
| 122 | 1 | CYA | UCC | UCC | VA3192 | KP769762 | 92.4 |
| 123 | 1 | CYA | Synechococcales | Synechococcus | VA3189 | KP769761 | 95.9 |
| 124 | 1 | OB | UCB | UCB | VA2182 | KP769713 | 77.4 |
| 125 | 1 | CYA | Synechococcales | Synechococcus | VA2188 | KP769714 | 94.9 |
| 126 | 1 | CYA | UCC | UCC | VA3185 | KP769760 | 92.7 |
| 127 | 1 | EP | UCE | UCE | VA21n16 | KP769717 | 88.6 |
| 128 | 1 | OB | UCB | UCB | VA1394 | NA | 91.1 |
| 129 | 1 | OB | UCB | UCB | VA22135 | NA | 83.2 |
| 130 | 1 | OB | Candidate | OD1 | VA22157 | KP769721 | 95.0 |
| 131 | 1 | OB | UCB | UCB | VA2215 | KP769720 | 88.4 |
| 132 | 1 | CYA | UCC | UCC | VA3170 | KP769758 | 87.3 |
| 133 | 1 | CYA | Pseudanabaenales | Pseudanabaena | VA221 | KP769718 | 94.6 |
| 134 | 1 | EP | Stramenopila |  | VA22309 | KP769724 | 92.4 |
| 135 | 1 | OB | Candidate | OD1-ZB2 | VA22319 | NA | 91.5 |
| 136 | 1 | OB | UCB | UCB | VA10101 | NA | 89.3 |
| 137 | 1 | OB | UCB | UCB | VA3158 | KP769757 | 97.1 |
| 138 | 1 | EP | Cryptophyta | Cryptophyta | VA2254 | KP769726 | 95.9 |
| 139 | 1 | OB | Candidate | OD1-ZB2 | VA1099 | KP769633 | 86.8 |
| 140 | 1 | OB | Chloroflexi | Ellin6529 | VA1066 | KP769621 | 97.1 |
| 141 | 1 | OB | UCB | UCB | VA1067 | KP769622 | 86.0 |
| 142 | 1 | CYA | Obscuribacterales | MLE-12 | VA106 | KP769618 | 94.0 |
| 143 | 1 | OB | Candidate | TM7-1 | VA1071 | KP769623 | 87.6 |
| 144 | 1 | OB | Candidate | OD1-ZB2 | VA1073n | NA | 91.2 |
| 145 | 1 | OB | Candidate | TM7-1 | VA1075 | KP769624 | 96.2 |
| 146 | 1 | OB | UCB | UCB | VA107n | KP769625 | 86.9 |
| 147 | 1 | OB | UCB | UCB | VA107 | NA | 81.1 |
| 148 | 1 | OB | UCB | UCB | VA1085 | NA | 84.7 |
| 149 | 1 | OB | UCB | UCB | VA1088 | KP769626 | 77.3 |
| 150 | 1 | OB | UCB | UCB | VA108 | NA | 89.7 |
| 151 | 1 | OB | Candidate | OD1 | VA1093n | KP769629 | 88.7 |
| 152 | 1 | OB | UCB | UCB | VA1093 | NA | 85.5 |
| 153 | 1 | EP | Stramenopila |  | VA1097 | KP769631 | 97.5 |
| 154 | 1 | OB | UCB | UCB | VA1098 | KP769632 | 84.9 |
| 155 | 1 | CYA | Synechococcales | Chamaesiphon | VA1064 | KP769620 | 94.3 |
| 156 | 1 | OB | UCB | UCB | VA109 | KP769627 | 83.0 |
| 157 | 1 | EP | UCE | UCE | VA1112 | KP769635 | 100.0 |
| 158 | 1 | OB | Candidate | TM7-1 | VA1116n | KP769636 | 93.6 |
| 159 | 1 | EP | UCE | UCE | VA1118n | KP769638 | 82.8 |
| 160 | 1 | CYA | Synechococcales | Cyanothece | VA1118 | KP769637 | 98.4 |
| 161 | 1 | CYA | UCC | UCC | VA1119 | KP769639 | 91.4 |
| 162 | 1 | CYA | Nostocales | Nostocaceae | VA1120 | KP769641 | 96.8 |
| 163 | 1 | CYA | Oscillatoriales | Phormidium | VA1121 | KP769642 | 97.2 |
| 164 | 1 | OB | Candidate | TM7-1 | VA1122 | KP769643 | 92.4 |
| 165 | 1 | EP | UCE | UCE | VA1123 | KP769644 | 91.1 |
| 166 | 1 | EP | UCE | UCE | VA1124 | KP769645 | 90.8 |
| 167 | 1 | EP | UCE | UCE | VA1126 | KP769647 | 93.4 |
| 168 | 1 | CYA | Pseudanabaenales | Pseudanabaenaceae | VA1127 | KP769648 | 95.6 |
| 169 | 1 | OB | Candidate | TM7-1 | VA1129 | KP769649 | 94.6 |
| 170 | 1 | CYA | Pseudanabaenales | Pseudanabaena | VA113n | KP769653 | 99.0 |
| 171 | 1 | OB | UCB | UCB | VA1026 | KP769607 | 81.6 |
| 172 | 1 | OB | UCB | UCB | VA10103 | KP769595 | 85.8 |
| 173 | 1 | CYA | Cyanobacteria | Cyanobacteria | VA10104 | KP769596 | 92.4 |
| 174 | 1 | OB | Candidate | OD1 | VA10105 | KP769597 | 87.7 |
| 175 | 1 | CYA | UCC | UCC | VA10108 | KP769598 | 81.6 |
| 176 | 1 | OB | Candidate | OD1-ZB2 | VA1011 | KP769599 | 92.4 |
| 177 | 1 | OB | Firmicutes | Anaerosinus | VA1013 | NA | 100.0 |
| 178 | 1 | OB | UCB | UCB | VA1015 | NA | 87.1 |
| 179 | 1 | OB | UCB | UCB | VA1016 | KP769600 | 84.9 |
| 180 | 1 | OB | UCB | UCB | VA1019 | KP769601 | 83.2 |
| 181 | 1 | OB | UCB | UCB | VA101 | KP769594 | 89.2 |
| 182 | 1 | EP | UCE | UCE | VA1021 | KP769603 | 91.4 |
| 183 | 1 | OB | Candidate | OD1 | VA1022 | KP769604 | 89.0 |
| 184 | 1 | OB | UCB | UCB | VA1024 | NA | 90.9 |
| 185 | 1 | OB | UCB | UCB | VA1025n | KP769606 | 86.4 |
| 186 | 1 | OB | UCB | UCB | VA1025 | KP769605 | 78.9 |
| 187 | 1 | CYA | Pseudanabaenales | Pseudanabaena | VA1092 | KP769628 | 94.2 |
| 188 | 1 | OB | UCB | UCB | VA1028 | KP769608 | 83.1 |
| 189 | 1 | EP | Cryptophyta |  | VA102 | KP769602 | 95.9 |
| 190 | 1 | OB | UCB | UCB | VA103n | KP769611 | 83.3 |
| 191 | 1 | OB | UCB | UCB | VA1035n | NA | 83.3 |
| 192 | 1 | OB | UCB | UCB | VA1036 | KP769610 | 88.9 |
| 193 | 1 | OB | UCB | UCB | VA1037 | NA | 82.1 |
| 194 | 1 | OB | UCB | UCB | VA1041 | NA | 90.0 |
| 195 | 1 | OB | UCB | UCB | VA1042 | KP769612 | 86.7 |
| 196 | 1 | OB | UCB | UCB | VA1046 | NA | 86.1 |
| 197 | 1 | OB | UCB | UCB | VA1048 | NA | 88.3 |
| 198 | 1 | EP | Streptophyta |  | VA1050n | KP769615 | 97.9 |
| 199 | 1 | OB | UCB | UCB | VA1053 | KP769616 | 91.7 |
| 200 | 1 | OB | UCB | UCB | VA1058 | KP769617 | 77.5 |
| 201 | 1 | OB | UCB | UCB | VA105 | KP769614 | 88.3 |
| 202 | 1 | OB | UCB | UCB | VA1062n | KP769619 | 90.2 |
| 203 | 1 | EP | Chlorophyta |  | VA1159 | KP769660 | 94.6 |
| 204 | 1 | CYA | Oscillatoriales | Phormidium | VA1134 | KP769651 | 99.1 |
| 205 | 1 | OB | Candidate | TM7-1 | VA1130 | KP769650 | 96.2 |
| 206 | 1 | CYA | Chroococcales | Xenococcaceae | VA1136 | KP769652 | 91.1 |
| 207 | 1 | CYA | UCC | UCC | VA1161 | KP769662 | 89.5 |
| 208 | 1 | OB | Candidate | TM7-1 | VA113nn | KP769654 | 96.2 |

^*^ Taxa were determined based on the taxonomies from Greengenes and Silva at ARB-Silva using SINA (v1.2.11) with the least common ancestor (LCA) method. Listed in the parentheses were from Silva taxonomy. UCC=unclassified cyanobacteria, UCE=unclassified eukaryotic phytoplankton, UCB= unclassified other bacteria. Blank cells are unclassified.
